# Petascale supercomputing to accelerate the design of high-temperature alloys

**DOI:** 10.1080/14686996.2017.1371559

**Published:** 2017-10-25

**Authors:** Dongwon Shin, Sangkeun Lee, Amit Shyam, J. Allen Haynes

**Affiliations:** ^a^ Materials Science and Technology Division, Oak Ridge National Laboratory, Oak Ridge, USA; ^b^ Computer Science and Mathematics Division, Oak Ridge National Laboratory, Oak Ridge, USA

**Keywords:** Supercomputing, first-principles calculations, density functional theory, correlation analysis, machine learning, alloys, 10 Engineering and Structural materials, 401 1st principle calculations, 404 Materials informatics / Genomics

## Abstract

Recent progress in high-performance computing and data informatics has opened up numerous opportunities to aid the design of advanced materials. Herein, we demonstrate a computational workflow that includes rapid population of high-fidelity materials datasets via petascale computing and subsequent analyses with modern data science techniques. We use a first-principles approach based on density functional theory to derive the segregation energies of 34 microalloying elements at the coherent and semi-coherent interfaces between the aluminium matrix and the *θ*′-Al_2_Cu precipitate, which requires several hundred supercell calculations. We also perform extensive correlation analyses to identify materials descriptors that affect the segregation behaviour of solutes at the interfaces. Finally, we show an example of leveraging machine learning techniques to predict segregation energies without performing computationally expensive physics-based simulations. The approach demonstrated in the present work can be applied to any high-temperature alloy system for which key materials data can be obtained using high-performance computing.

## Graphical abstract

**Figure:**
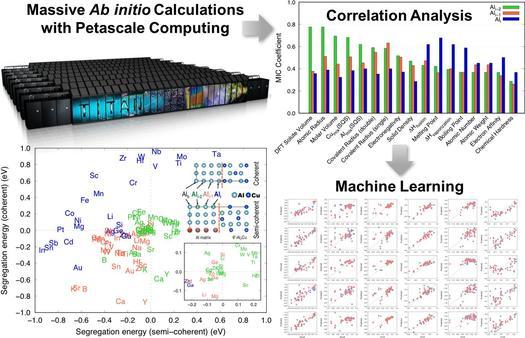

## 1. Introduction

A recent Oak Ridge National Laboratory (ORNL) study by Shyam et al. [1] demonstrated that it is possible to exert a remarkable stabilizing influence on thermodynamically metastable strengthening precipitates (*θ*′-Al_2_Cu) within aluminium-copper (Al-Cu) alloys to temperatures of at least 300 °C via microalloying with manganese (Mn) and zirconium (Zr). An extensive experimental investigation using atom probe tomography and scanning transmission electron microscopy revealed that trace amounts of microalloyed solute atoms segregated at the interfaces and inhibited the coarsening process that leads to detrimental phase transformation (*θ*′→*θ*) at elevated temperatures. This exciting experimental observation of an extended temperature regime of precipitate stability offers numerous opportunities to design new classes of alloys with significantly improved mechanical properties at high temperatures by understanding and then manipulating the interfacial stability of key precipitates.

Shyam et al. [1] also showed that a hierarchy and synergy exist in terms of the ability of individual or combined elements to stabilize the key interfaces in *θ*′ precipitates at high temperatures. They demonstrated that either Mn or Zr additions alone provide stabilization of *θ*′ only to a certain extent, but the critical temperature – below which stability and strength can be preserved – can be further elevated by adding Mn and Zr together. The underlying origin of the observed elemental hierarchy, which would be extremely useful for high-temperature alloy design, is currently unknown. Identifying such favourable combinations of microalloying elements solely via experimental studies would be prohibitively time consuming and costly because of the complex multi-component nature of most high-temperature alloys. Accurate theoretical guidance and prediction of the effects of microalloying additions will have an across-the-board impact on the understanding of the mechanical behaviour of the next generation of high-temperature alloys.

It has been steadily demonstrated during the past couple of decades that first-principles density functional theory (DFT) calculations can provide accurate predictions of energetics, diffusion kinetics, and lattice mismatch/strain in alloys [2]. However, most of these type DFT calculations have been focused on elucidating the underlying physics and chemistry in single-phase materials. To directly guide the design of multi-phase high-temperature alloys, more complex models that can better represent realistic materials systems and key interfaces are needed. Usually, such models are supercells with a large number of atoms – of the order of a hundred atoms – and the size of supercells has often been limited by the available computing power. In addition, constructing a large DFT database is desirable to identify element(s) that can promote improved physical/chemical properties of alloys. However, performing a series of DFT calculations of large supercells in a high-throughput manner within a reasonable timeframe requires an extensive amount of computing resources in a short period. Moreover, analysing large datasets populated from massive physics-based calculations to generate materials hypotheses is also a daunting task.

In the current work, we used a defect supercell approach in a high-throughput manner to construct a large first-principles database of the segregation energies of 34 microalloying solutes at both coherent and semi-coherent interfaces between an Al matrix and *θ*′-Al_2_Cu. We then performed correlation analyses to investigate key materials descriptors that govern the segregation behaviour of solute atoms at the interfaces. Finally, we demonstrated a theoretical framework that can predict segregation energies of solutes using machine learning techniques. The theoretical workflow introduced herein can be applied to any system where high-fidelity datasets can be rapidly populated from high-throughput calculations followed by extensive correlation analyses. The hypothesis and knowledge base generated in the demonstrated workflow are examples of how the design of some classes of advanced materials can be computationally guided or even led.

## 2. Computational approaches

### 2.1. First-principles calculations

#### 2.1.1. Solute segregation energy

We used a supercell approach to simulate the solute segregated coherent and semi-coherent interfaces between the Al matrix and *θ*ʹ-Al_2_Cu precipitate after Biswas et al. [3]. The pure FCC Al and *θ*ʹ-Al_2_Cu phases were fully relaxed, respectively, before being combined to create supercell models for coherent and semi-coherent interfaces. Next, the two generated supercells were fully relaxed with a periodic boundary condition invoked in all directions to induce a strain effect between the two phases. Finally, when solute atoms were introduced within two supercells, the supercell was forced to retain its volume and shape, while the internal positions of individual atoms were allowed to be relaxed. In addition, the periodic boundary condition was only invoked in the directions normal to the long axis of the supercell.

**Figure 1.**
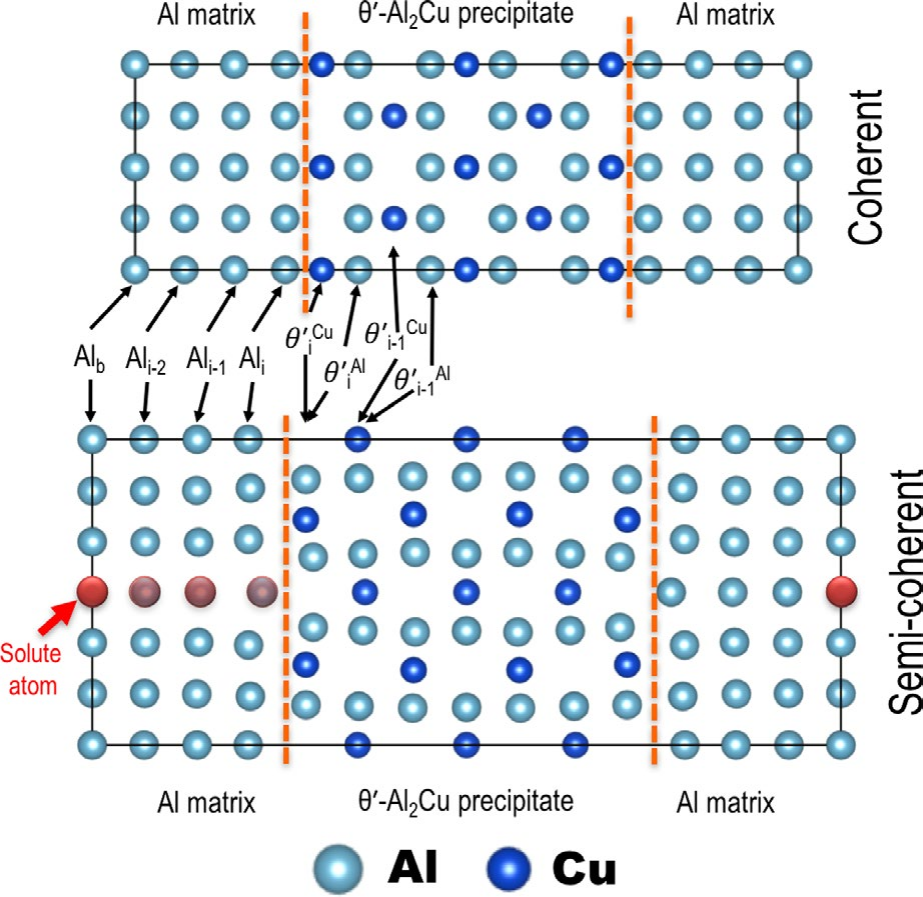
Relaxed supercells used for the Al/*θ*ʹ interfacial segregation calculations. Vertical lines in each figure represent interfaces. Top: coherent interface (108 atoms); bottom: semi-coherent interface (168 atoms). The red atom (indicated with an arrow on the fourth row) in the semi-coherent supercell shows the position of a solute at a ‘bulk’ site in the Al matrix, which is sufficiently far from the interface. Platelets are labelled according to their positions relative to the interface planes. See the text for details.

We considered the segregation of 34 elements; the supercell models are illustrated in Figure 1. Only one solute was introduced in each supercell, and a solute was progressively moved from the bulk toward the interface to derive the solute segregation energy as a function of the location from the interface. We considered all the crystallographically distinctive lattice sites at each platelet. As shown in Figure 1, four platelets were used to represent the Al matrix at both interfaces. They are denoted as Al_*x*_, where *x* represents the locations of solutes, *i* the interface, and *i*–1 and *i*–2 the first and second platelets away from the interface on the matrix side. The Al_*i*–3_ platelet was assumed to be sufficiently far away from the interface to be considered ‘bulk’ and was denoted Al_*b*_.

The solute segregation energy can be described by the difference between the two total energies as shown in equation (1):(1)
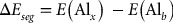



where *E* represents the total energy from first-principles DFT electronic structure calculations. The details of the DFT approach to deriving the segregation energies of solutes at the interfaces between Al and *θ*ʹ-Al_2_Cu are presented in Ref. [4].

#### 2.1.2. Petascale supercomputing

**Figure 2.**
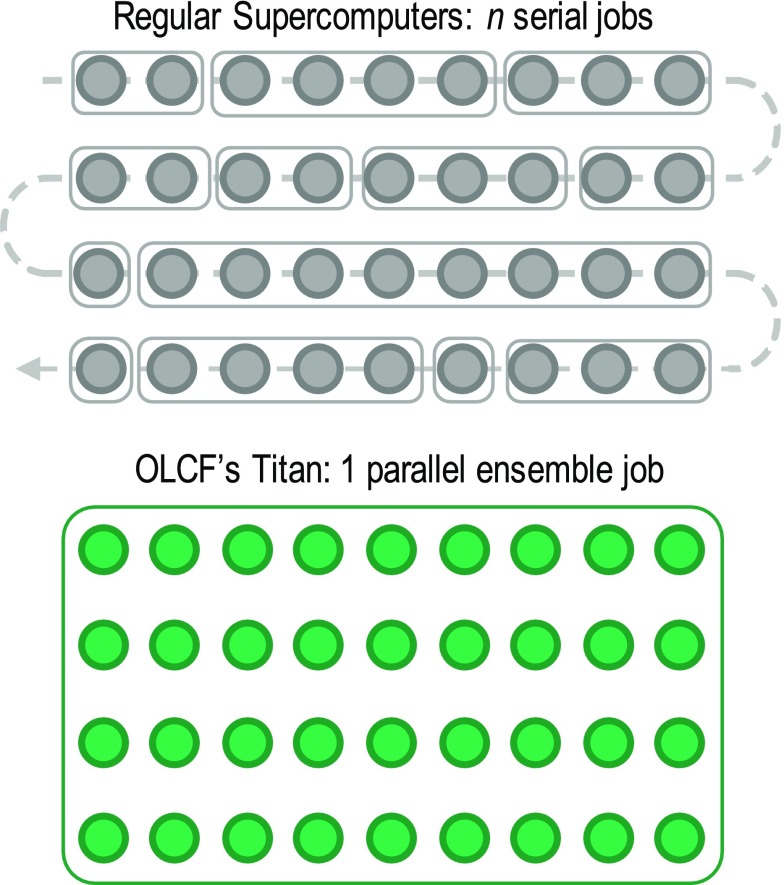
Each circle represents an individual supercell calculation. Titan at Oak Ridge Leadership Computing Facility (OLCF) allows the creation of an ensemble job to run many DFT calculations in parallel.

Eighteen supercell calculations (5 and 13 supercells for coherent and semi-coherent interfaces, respectively, to consider crystallographically distinctive sites in each platelet) per element were required to derive solute segregation with the DFT approach described above. Thus, several hundred DFT supercell calculations in total were required to construct a comprehensive solute segregation energy database to consider 34 elements. If regular high-performance computing (HPC) resources had been used to run the several hundred supercell DFT calculations, the same number of jobs would have been submitted in serial as illustrated in Figure 2.

To accommodate many users on limited resources and to maximize efficiency, most computing centres have adopted job queueing systems based on a priority policy. In general, the more resources users request and spend, the sooner they lose their priority. Hence, a user may run several hundred calculations fast in the early stages by running multiple jobs simultaneously while the priority is high. However, the progress will slow down as the accumulated usage becomes larger, until it reaches a periodic priority reset process. Although the computing power and capacity of currently available HPC resources have increased steadily over the past decade, the demand for access to supercomputing resources also has increased exponentially. Thus, implementing a large number of expensive calculations on regular HPC resources has become even more challenging in recent years.

In this regard, the leadership computing facilities of the US Department of Energy (DOE) offer unique opportunities by supporting computationally intensive large-scale projects with large amounts of dedicated time on supercomputers. The Cray XK7 supercomputer Titan, hosted at the Oak Ridge Leadership Computing Facility (OLCF), has 18,688 compute nodes that offer a peak performance of around 20 petaflops. As a DOE Leadership Computing Facility, the OLCF has a mandate that a large portion of Titan’s computing time be allocated to exceptionally large jobs. To ensure the OLCF complies with DOE directives, users are encouraged to run jobs on Titan that are as large as their code will warrant, as shown in Table 1. As illustrated in Figure 2, several hundred supercell calculations can be grouped together as a number of ensemble jobs. This unique allocation policy at OLCF enables researchers to tackle problems in which extensive theoretical calculations can provide insight into the design and development of advanced materials.

**Table 1.** Job priority by node count at the Oak Ridge Leadership Computing Facility, which implements queue policies that enable large jobs to run in a timely fashion.

Bin	Min nodes	Max nodes	Max walltime (hours)
1	11,250	–	24.0
2	3,750	11,249	24.0
3	313	3,749	12.0
4	126	312	6.0
5	1	125	2.0

In addition, we took advantage of the graphic processing unit (GPU) hybrid architecture of Titan and exploited the GPU version of the widely used DFT code Vienna Ab initio Simulation Package (VASP) [5,6], which offers ~2–3× faster performance than its counterpart CPU version. Currently, GPU-VASP supports only real-space projection, but extensive benchmark calculations against the CPU-VASP code supporting reciprocal-space projection have shown that the difference in the solute segregation energy is less than 0.1 meV for the current supercell calculations. All the DFT supercell calculations were performed using projector augmented wave [7] potentials and the generalized gradient approximation [8]. We used Perdew-Burke-Ernzerhof for the exchange–correlation functional [9].

#### 2.1.3. Materials descriptors from DFT calculations

We included three DFT-derived materials descriptors in the correlation analysis: DFT solute volume and the mixing energies in the Al and Cu sublattices within *θ*′-Al_2_Cu via special quasi-random structures (SQSs) [10]. We calculated the solute volume within Al by comparing the DFT volume difference with and without a solute in the Al supercell, as described in previous studies [11,12]. Al_108_ (3 × 3×3) and Al_107_X supercells were used to obtain the solute volumes corresponding to 34 elements. SQSs can mimic random mixing between solute atoms and the respective Al/Cu sublattices of *θ*′-Al_2_Cu by satisfying the atomic arrangement quantified as correlation functions. Therefore, SQSs can serve as structural templates to derive the mixing energies by switching atomic identities within the context of DFT electronic structure calculations. SQS mixing energies in the Al and Cu sublattices are denoted as Al_mix_(SQS) and Cu_mix_(SQS), respectively, hereafter.

### 2.2. Materials informatics

#### 2.2.1. Correlation Analysis

Using the generated DFT database of solute segregation energies in Al-Cu alloys, we investigated the results to identify key descriptors by performing correlation analysis. We analysed the segregation energies of 34 elements in both coherent and semi-coherent interfaces with 17 atomistic descriptors in five different groups:

• atomic size/volume: atomic number, weight, radius, DFT solute volume, molar volume and covalent radius (single and double bond);
• atomic structure: electron affinity;
• physical property: density at 25 °C;
• atomic interaction: Pauling electronegativity and chemical hardness;
• thermodynamic properties: melting point, boiling point, enthalpy of fusion (Δ*H*_fusion_), enthalpy of vaporization (Δ*H*_vaporization_), and SQS mixing energies in Al and Cu sublattices denoted as Al_mix_(SQS) and Cu_mix_(SQS), respectively.

In analysing the dataset for the DFT solute segregation energy combined with the 17 atomistic descriptors, we applied an advanced technique called maximal information coefficient (MIC) analysis [13], as well as conventional Pearson’s correlation coefficient (PCC) analysis. We used both the MIC and PCC approaches to crosscheck whether the identified descriptors are consistent and to select features to be used to train machine learning models.

#### 2.2.2. Machine learning

We explored the feasibility of developing data-driven approaches, such as data mining and machine learning, to predict segregation energies without performing computing-intensive tasks (e.g. DFT and molecular dynamic simulations). The numerical approach to predicting output variable value for given input variables by estimating the relationship between input variables and output variables is referred to as regression in the machine learning technique. We used a Python-based open source data analytics toolkit, *scikit*-*learn* [14], which contains a wide variety of machine learning algorithms. The library classifies regression models in several categories such as *generalized linear models*, *Kernel ridge regression*, *nearest neighbours*, *ensemble methods*, etc. To cover the variety of different machine learning models, we picked two from *generalized linear models* (Linear regression [15], Bayesian ridge regression [16]), *Kernel ridge regression* [17] (itself a separate category), one from *nearest neighbours* (Nearest neighbour regression [18]), and one from *ensemble methods* (Random forest regression [19]). A brief introduction to the individual machine learning models used in the current study is presented below.

*Linear regression* models the relationships between input and output variables using a linear predictor function, a linear model, and fits to minimize the residual sum of squares between the observed data values and predicted values by the linear approximation.

*Bayesian Ridge regression* is another linear regression model, but it takes a probabilistic approach. It estimates optimal parameter values for a probabilistic model, assuming the output variable follows a normal distribution. To overcome the overfitting, based on Bayesian inference, it incorporates prior distributions for the estimated parameters for the model.

*Kernel Ridge regression* is a model to estimate the conditional expectation of a random variable to find a non-linear relation between a pair of random variables. It uses the kernel method for simplifying the computation of inner products in a high-dimensional space and learns a linear model in the implicit feature space induced by a kernel and the dataset.

*(k-)Nearest neighbours regression* is a non-parametric method that can be used when the input variables are continuous variables. It is one of the simplest machine learning algorithms, as it simply outputs the average of the values of given data points, *k* nearest neighbours. It is considered to be a lazy learning method, as it defers the computation and uses only a portion of relevant datasets.

*Random forest regression* is an ensemble learning method that constructs multiple decision trees; it learns recursive decision rules inferred from the data features represented as a tree structure at training time and outputting mean prediction of the individual trees. Using multiple decision trees is one technique to overcome the decision tree’s overfitting to the training dataset.

We arbitrarily selected the top ten ranking features to be used within the model training based on our correlation analyses. The DFT solute segregation database generated in the current work was used as an input dataset for training these machine learning models.

## 3. Results and discussion

### 3.1. DFT solute segregation energies

**Figure 3.**
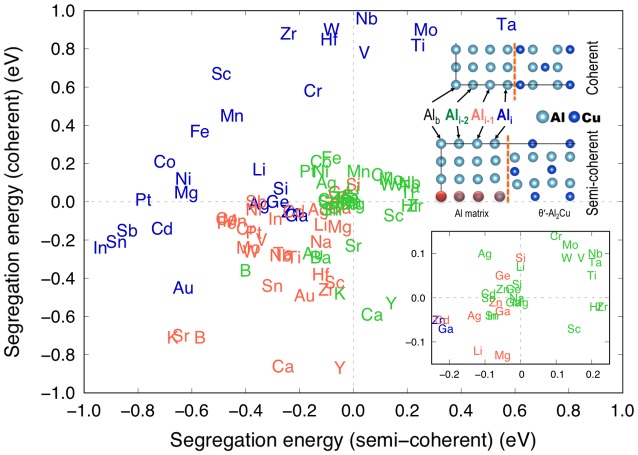
DFT segregation energies of 34 solutes at each platelet (Al_*i*–2_, Al_*i*–1_ and Al_*i*_) at the coherent and semi-coherent interfaces between the Al matrix and *θ*′-Al_2_Cu. Only the lowest segregation energies at given platelets are shown.

Solute segregation energies at both the coherent and semi-coherent interfaces at each platelet are plotted in Figure 3. This plot can be categorized into four quadrants, which combine favourable and unfavourable solute segregation as negative and positive segregation energies, respectively, at the two different interfaces. Notably, both Mn and Zr belong in the upper left quadrant in which segregation is favourable at the semi-coherent but not at the coherent interface at Al_*i*_. Scandium (Sc), which has been experimentally reported to stabilize *θ*′ by solute segregation and thus provide high coarsening resistance at high temperatures [20,21], also belongs to the same quadrant. Therefore, we can correlate the improved high-temperature stability of Al-Cu alloys to microalloying elements (Mn, Zr, and Sc) and their anisotropic solute segregation energies at the two different types of interfaces (and along various platelet positions) of *θ*′ precipitates. Based on the observed relationship here, we may propose the use of microalloying elements other than the ones that already have been experimentally verified to improve the high-temperature strength and stability of cast Al-Cu alloys.

In selecting microalloying elements, one can consider a strategy of adding multiple elements to create a synergistic effect. Shyam et al*.* [1] showed experimentally that there is are evident positive (as well as other negative) synergies in microalloying with more than one element to stabilize *θ*′ at elevated temperatures, and a hierarchy exists in such combinations. It was demonstrated that either Mn or Zr additions alone could provide stabilization of *θ*′ only to a certain extent; however, the critical temperature to which *θ*′ can be stabilized can significantly be elevated by adding both Mn and Zr, while also limiting or eliminating other elements. Hence, it would be worthwhile to experimentally test combinations of elements in the first quadrant in which Mn and Zr are located. A detailed discussion on identifying microalloying elements to *θ*ʹ-Al_2_Cu at high temperatures in conjunction with the physical mechanisms associated with the DFT segregation energy database is provided in Ref. [4].

### 3.2. Correlation analysis

**Figure 4.**
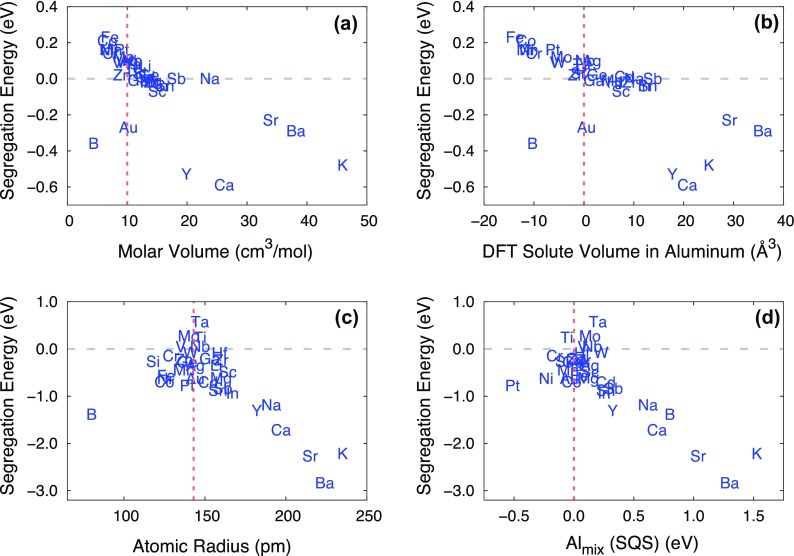
DFT solute segregation energies plotted with selected descriptors: (a) molar volume at the coherent interface (Al_*i*–2_); (b) DFT solute volume in Al at the coherent interface (Al_*i*–2_); (c) atomic radius at the semi-coherent interface (Al_*i*_); and (d) mixing energy of solute at the Al sublattice in *θ*′-Al_2_Cu from DFT SQSs calculation at the semi-coherent interface (Al_*i*_). Vertical lines in each figure represent the respective values for Al, while the DFT solute volume of Al itself and the mixing within the Al sublattice in *θ*′-Al_2_Cu are treated as zero.

We started by plotting all 17 descriptors with respect to the DFT segregation energies at three different platelets, Al_*i*_, Al_*i*–1_, and Al_*i*–2_, at both the coherent and semi-coherent interfaces for visual analysis. Selected plots are shown in Figure 4. We identified a linear relationship between the DFT solute segregation energies and some select descriptors. Notably, these linear relationships were dominant at Al_*i*–2_ at the coherent interface and Al_*i*_ at the semi-coherent interface.

**Figure 5.**
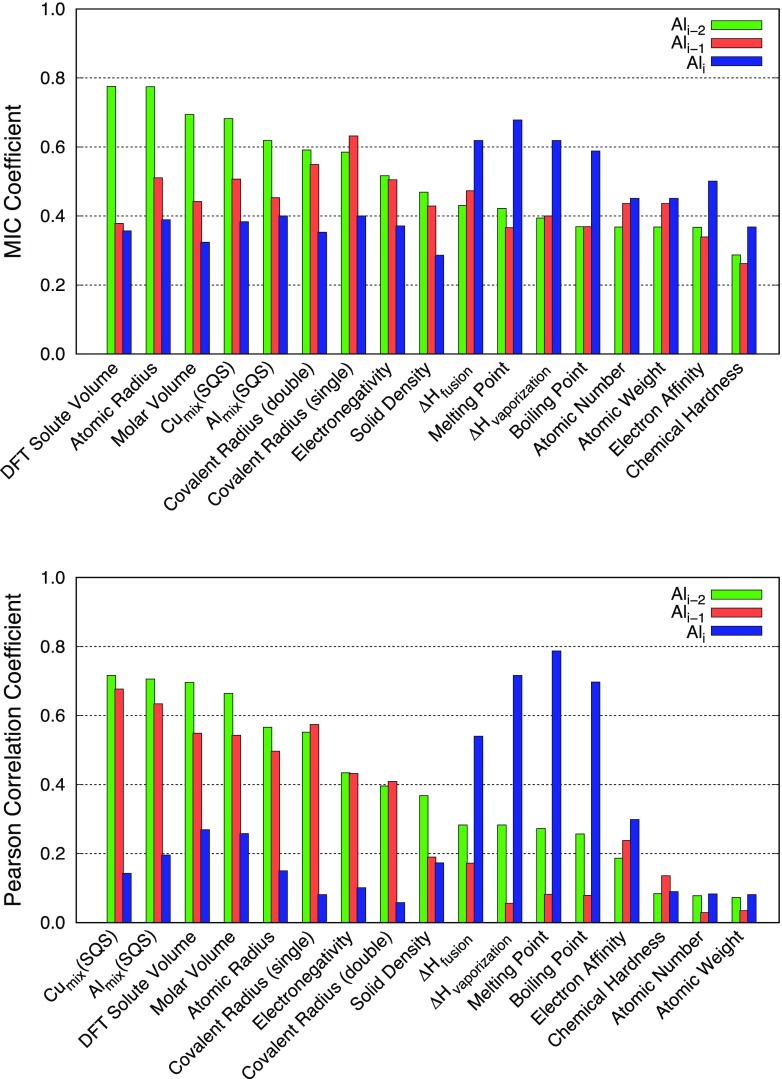
Correlation coefficients between the solute segregation energies derived from DFT calculations at the coherent interface (Al_*i*_, Al_*i*–1_, and Al_*i*–2_ platelets) and materials descriptors with MIC (top) and Pearson (bottom) approaches.

**Figure 6.**
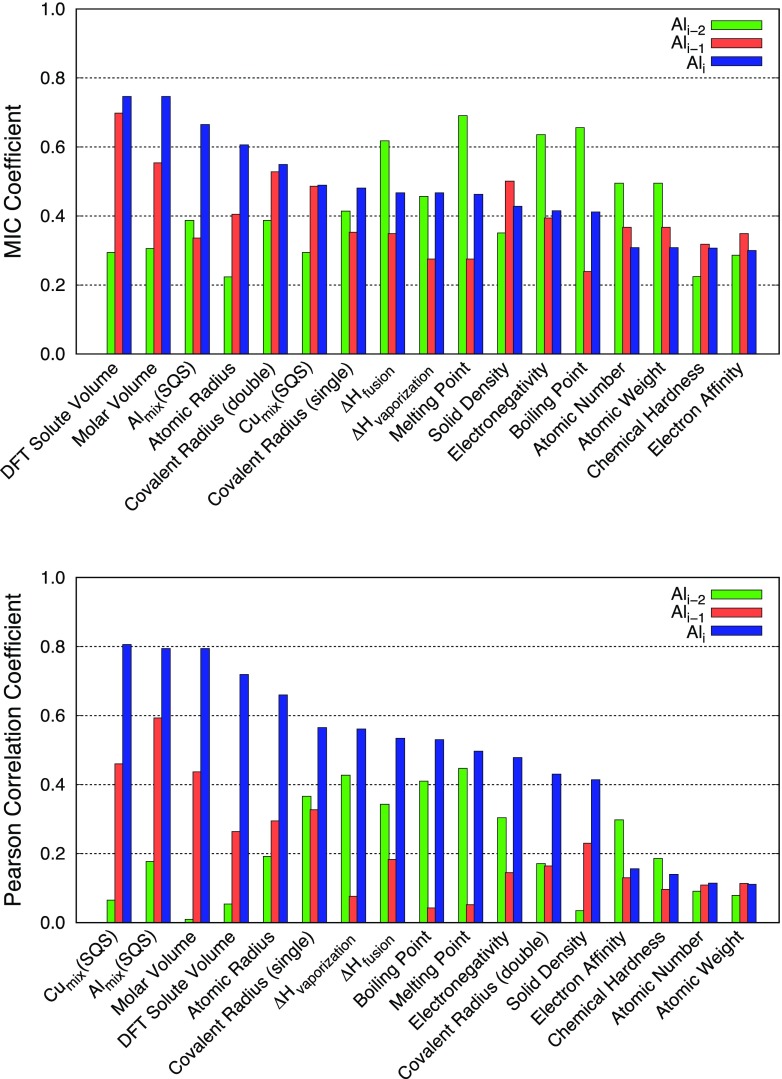
Correlation coefficients between the solute segregation energies derived from DFT calculations at the semi-coherent interface (Al_*i*_, Al_*i*–1_, and Al_*i*–2_ platelets) and materials descriptors with MIC (top) and Pearson (bottom) approaches.

Afterward, we performed more systematic correlation and regression analyses to quantify the relationship between the solute segregation energies and descriptors at both coherent and semi-coherent interfaces using the MIC and PCC approaches. Overall, the two different correlation analysis methods were in good agreement with each other (see Figures 5 and 6). Both the MIC and PCC approaches found that size- and volume-related descriptors (e.g. molar volume, atomic radius and DFT volume) are strongly correlated with solute segregation at both interfaces. A similar size effect – i.e. that large solutes tend to bind more strongly with vacancies in Al [11] and Mg [12], respectively – has previously been reported in DFT studies. This observation shows that a size argument can also be applied in solute segregation.

On the other hand, some elements exhibited a tendency to be segregated from the interface, even though they were smaller in size/volume than Al at the semi-coherent interface. This observation suggests that the size/volume effect is important, but it alone cannot fully explain the segregation behaviour of solute atoms at the semi-coherent interface. For example, solubilities of microalloying elements within *θ*′, represented as mixing energies from SQS calculations in the Al and Cu sublattices (Al_mix_(SQS) and Cu_mix_(SQS)), are strongly correlated with solute segregation energies. As shown in Figures 5 and 6, both MIC and PCC methods identified that size, and solubility-related descriptors (e.g. atomic radius, volume, DFT solute volume and SQSs) are strongly correlated with the segregation at Al_*i*–2_ in the coherent and Al_*i*_ in the semi-coherent interface, respectively.

### 3.3. Machine learning

**Figure 7.**
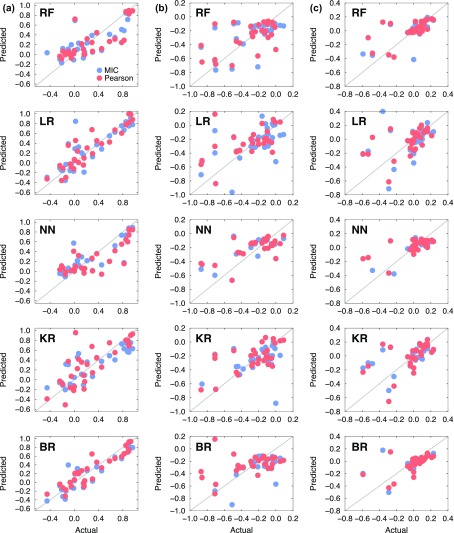
Scatter plots for the five machine learning models (RF: random forest, LR: linear regression, NN: nearest neighbour, KR: kernel ridge, BR: Bayesian ridge). The *x*- and *y*-axes represent the acutal and predicted solute segregation energies of 34 elements at the coherent interfaces (a) Al_*i*_, (b) Al_*i*–1_, and (c) Al_*i*–2_.

**Figure 8.**
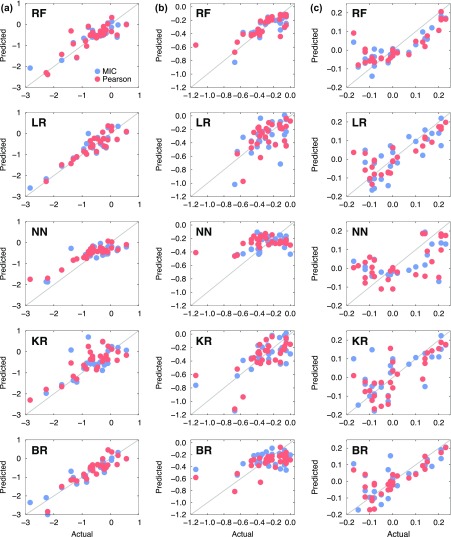
Scatter plots for the five machine learning models (RF: random forest, LR: linear regression, NN: nearest neighbour, KR: kernel ridge, BR: Bayesian ridge). The *x*- and *y*-axes represent the acutal and predicted solute segregation energies of 34 elements at the semi-coherent interfaces (a) Al_*i*_, (b) Al_*i*–1_, and (c) Al_*i*–2_.

Choosing appropriate features to be included in the training of machine learning models can be of its own topic (feature selection). Our intention in the current work is to introduce an emerging computational approach to the structural materials community, rather than how to exercise it in detail. Hence, we arbitrarily selected ten top ranking features out of 17 from both the MIC and PCC analyses and used them to train the machine learning models. The performance of the correlation analyses and the accuracy of the machine learning models are summarized in Table 2. Scattered plots comparing the actual and predicted segregation energies of the coherent and semi-coherent interfaces are presented in Figures 7 and 8.

**Table 2.** Comparison of the performance of two different correlation analyses (MIC and Pearson) and the corresponding five different machine learning models at each platelet in the coherent and semi-coherent interface. Higher values of *R*/*R* ^2^ and lower values of root-mean-square error (RMSE) represent better accuracy.

Platelets	Values	Correlation analysis	Machine learning models				
			RF	LR	NN	KR	BR
Coherent(Al_i_)	*R*	MIC	0.799	0.856	0.820	0.842	0.920
		Pearson	0.785	0.909	0.804	0.786	0.923
	*R*^2^	MIC	0.639	0.733	0.672	0.709	0.847
		Pearson	0.616	0.826	0.646	0.618	0.851
	RMSE	MIC	0.239	0.227	0.224	0.238	0.176
		Pearson	0.248	0.173	0.249	0.269	0.155
Coherent(Al_*i*–1_)	*R*	MIC	0.574	0.564	0.710	0.429	0.359
		Pearson	0.572	0.505	0.672	0.594	0.398
	*R*^2^	MIC	0.329	0.318	0.505	0.184	0.129
		Pearson	0.328	0.255	0.452	0.353	0.158
	RMSE	MIC	0.213	0.233	0.180	0.264	0.256
		Pearson	0.233	0.259	0.192	0.227	0.253
Coherent(Al_*i*–2_)	*R*	MIC	0.757	0.408	0.800	0.535	0.550
		Pearson	0.631	0.509	0.666	0.526	0.629
	*R*^2^	MIC	0.573	0.167	0.640	0.286	0.302
		Pearson	0.399	0.259	0.443	0.277	0.396
	RMSE	MIC	0.127	0.228	0.108	0.193	0.146
		Pearson	0.154	0.187	0.144	0.193	0.141
Semi-coherent(Al_*i*_)	*R*	MIC	0.886	0.909	0.788	0.683	0.910
		Pearson	0.846	0.908	0.918	0.834	0.886
	*R*^2^	MIC	0.786	0.825	0.621	0.467	0.829
		Pearson	0.715	0.824	0.843	0.695	0.785
	RMSE	MIC	0.334	0.309	0.410	0.484	0.327
		Pearson	0.354	0.314	0.374	0.456	0.295
Semi-coherent(Al_*i*–1_)	*R*	MIC	0.786	0.575	0.531	0.694	0.406
		Pearson	0.793	0.609	0.475	0.674	0.669
	*R*^2^	MIC	0.618	0.331	0.282	0.481	0.165
		Pearson	0.629	0.371	0.226	0.455	0.448
	RMSE	MIC	0.107	0.198	0.153	0.192	0.209
		Pearson	0.151	0.169	0.221	0.194	0.186
Semi-coherent(Al_*i*–2_)	*R*	MIC	0.843	0.784	0.585	0.681	0.608
		Pearson	0.531	0.858	0.579	0.793	0.783
	*R*^2^	MIC	0.710	0.614	0.342	0.463	0.370
		Pearson	0.282	0.736	0.335	0.629	0.614
	RMSE	MIC	0.067	0.093	0.117	0.095	0.118
		Pearson	0.123	0.075	0.105	0.085	0.080

Overall, the quality of prediction was satisfactory only in select cases. This is mainly attributable to the lacking instances of 34 elements in each platelet (Al_*i*_, Al_*i*–1_, and Al_*i*–2_ at both the coherent and semi-coherent interfaces). However, the agreement between the actual and predicted segregation energies was quite good even with the limited datasets. For example, most of the machine learning models predicted the segregation energies quite reliably regardless of the correlation analyses at the Al_*i*_ platelet of the semi-coherent interface, as shown in Figure 8(a). The machine learning predictions at the Al_*i*_ platelet of the coherent interface, as shown in Figure 7(a), were also quite good. Remarkably, Al_*i*_ platelets at both coherent and semi-coherent interfaces were analysed as having strong correlations with the respective descriptors, as shown in Figures 5 and 6 (see the third bars of each descriptor in both figures). It is encouraging that reliable data-driven predictive models can be developed in an automated fashion even with a relatively small number of pure theoretical calculations, if strong correlations exist between specific descriptors and materials properties. With recent progress in the data science and open source communities, advanced data analytics tools are now readily available to researchers in other domains. Although successful prediction of segregation energies is limited to a small number of cases, the authors contend that the presented workflow has tremendous potential for application in design and development of improved high-temperature alloys.

## 4. Conclusions

We considered a total of 34 elements as potential microsegregants to improve the stability of *θ*′-Al_2_Cu at elevated temperatures within the framework of high-throughput DFT supercell calculations. Several hundred defect supercells were computed in an ensemble to take advantage of the petascale computing resources at the OLCF. The populated large DFT dataset in the current work was fused with various atomistic descriptors and interrogated to reveal correlations. The correlation analysis results were used to select features to be used in the training of machine learning models. Even with a small training dataset, the select machine learning models developed could reliably predict segregation energies. The accuracy of machine learning models may be further improved by incorporating an expanded DFT dataset of other alloying elements and considering more atomistic features, which may have higher correlations with segregation energies. The computational workflow demonstrated in the current work can be applied to accelerate the design of other high-temperature alloy systems in which high-fidelity datasets can be rapidly populated via supercomputer calculations followed by modern data analytics.

## Disclosure statement

No potential conflict of interest was reported by the authors.

## Supplemental data

The supplemental material for this paper is available online at https://doi.org/10.1080/14686996.2017.1371559

## Supplementary Material

Supplementary.docx
