# Supplementary material for: Petascale supercomputing to accelerate the design of high-temperature alloys
Source: Sci Technol Adv Mater. 2017 Oct 25;18(1):828–38. doi: 10.1080/14686996.2017.1371559 (PMC5782487; doi:10.1080/14686996.2017.1371559)
Supplement: Supplementary.docx [file TSTA_A_1371559_SM1825.docx]

Table 1 Solute segregation energies at the coherent and semi-coherent interfaces between Al and 𝜃′ from first-principles density functional theory calculations presented in Figure 3.

| Element | Segregation energy (eV/solute) | | | | | |
| --- | --- | --- | --- | --- | --- | --- |
|  | Coherent | | | Semi-coherent | | |
|  | Al_i-2_ | Al_i-1_ | Al_i_ | Al_i-2_ | Al_i-1_ | Al_i_ |
| Li | 0.07 | -0.12 | 0.17 | 0.00 | -0.12 | -0.35 |
| B | -0.36 | -0.71 | 0.02 | -0.40 | -0.57 | -1.39 |
| Na | 0.00 | -0.21 | 0.16 | -0.01 | -0.12 | -1.19 |
| Mg | -0.01 | -0.13 | 0.05 | 0.00 | -0.05 | -0.62 |
| Si | 0.03 | 0.09 | 0.07 | -0.01 | 0.00 | -0.27 |
| K | -0.48 | -0.71 | -0.19 | -0.05 | -0.67 | -2.22 |
| Ca | -0.59 | -0.86 | -0.10 | 0.07 | -0.26 | -1.72 |
| Sc | -0.07 | -0.42 | 0.67 | 0.15 | -0.07 | -0.49 |
| Ti | 0.05 | -0.29 | 0.82 | 0.20 | -0.22 | 0.24 |
| V | 0.09 | -0.20 | 0.78 | 0.17 | -0.34 | 0.04 |
| Cr | 0.14 | -0.14 | 0.58 | 0.10 | -0.40 | -0.15 |
| Mn | 0.16 | -0.10 | 0.45 | 0.02 | -0.44 | -0.45 |
| Fe | 0.23 | -0.11 | 0.37 | -0.08 | -0.47 | -0.57 |
| Co | 0.21 | -0.09 | 0.21 | -0.12 | -0.47 | -0.70 |
| Ni | 0.16 | -0.04 | 0.12 | -0.12 | -0.37 | -0.63 |
| Cu | 0.08 | 0.01 | 0.04 | -0.07 | -0.18 | -0.41 |
| Zn | 0.02 | -0.01 | -0.05 | -0.05 | -0.07 | -0.23 |
| Ga | -0.01 | -0.03 | -0.07 | -0.02 | -0.05 | -0.21 |
| Ge | 0.02 | 0.05 | 0.00 | -0.02 | -0.05 | -0.28 |
| Sr | -0.23 | -0.70 | 0.30 | 0.00 | -0.64 | -2.27 |
| Y | -0.53 | -0.87 | 0.29 | 0.14 | -0.05 | -1.31 |
| Zr | -0.02 | -0.46 | 0.88 | 0.23 | -0.10 | -0.24 |
| Nb | 0.10 | -0.28 | 0.96 | 0.21 | -0.27 | 0.05 |
| Mo | 0.12 | -0.24 | 0.90 | 0.14 | -0.39 | 0.27 |
| Ag | 0.10 | -0.04 | -0.01 | -0.10 | -0.13 | -0.35 |
| Cd | 0.01 | -0.05 | -0.14 | -0.09 | -0.22 | -0.71 |
| In | -0.04 | -0.09 | -0.24 | -0.08 | -0.29 | -0.94 |
| Sn | -0.04 | -0.44 | -0.21 | -0.08 | -0.30 | -0.88 |
| Sb | 0.00 | 0.00 | -0.15 | -0.09 | -0.36 | -0.84 |
| Ba | -0.29 | -0.51 | 0.15 | -0.12 | -1.14 | -2.84 |
| Hf | -0.02 | -0.38 | 0.85 | 0.21 | -0.12 | -0.09 |
| Ta | 0.08 | -0.27 | 0.93 | 0.21 | -0.26 | 0.57 |
| W | 0.09 | -0.26 | 0.90 | 0.13 | -0.38 | -0.08 |
| Pt | 0.16 | -0.16 | 0.01 | -0.17 | -0.37 | -0.78 |
| Au | -0.27 | -0.49 | -0.45 | -0.15 | -0.18 | -0.63 |

Table 2 Atomistic descriptors of 34 elements presented in Figure 4 with DFT solute segregaton energies at the coherent and semi-coherent interfaces between Al and 𝜃′.

|  | Molar  Volume  (cm^3^/mol) | DFT Solute Volume (Å^3^) | Atomic Radius  (pm) | Al_mix_ (SQS)  (eV) |
| --- | --- | --- | --- | --- |
| Li | 13.02 | -0.54 | 157 | 0.09 |
| B | 4.39 | -10.30 | 80 | 0.80 |
| Na | 23.78 | 10.07 | 191 | 0.62 |
| Mg | 14.00 | 5.69 | 160 | 0.12 |
| Si | 12.06 | -1.28 | 118 | -0.10 |
| K | 45.94 | 25.04 | 235 | 1.53 |
| Ca | 26.20 | 20.73 | 197 | 0.69 |
| Sc | 15.00 | 7.41 | 164 | 0.13 |
| Ti | 10.64 | -0.90 | 147 | -0.06 |
| V | 8.32 | -5.85 | 135 | 0.07 |
| Cr | 7.23 | -10.00 | 129 | -0.16 |
| Mn | 7.35 | -11.31 | 137 | -0.05 |
| Fe | 7.09 | -13.82 | 126 | 0.04 |
| Co | 6.67 | -11.57 | 125 | -0.02 |
| Ni | 6.59 | -11.44 | 125 | -0.23 |
| Zn | 9.16 | -1.37 | 137 | 0.01 |
| Ga | 11.80 | 2.01 | 153 | 0.02 |
| Ge | 13.63 | 2.64 | 139 | -0.01 |
| Sr | 33.94 | 29.15 | 215 | 1.04 |
| Y | 19.88 | 17.71 | 182 | 0.32 |
| Zr | 14.02 | 9.11 | 160 | 0.08 |
| Nb | 10.83 | 0.21 | 147 | 0.15 |
| Mo | 9.38 | -4.51 | 140 | 0.13 |
| Ag | 10.27 | 1.58 | 144 | 0.13 |
| Cd | 13.00 | 8.10 | 152 | 0.27 |
| In | 15.76 | 12.07 | 167 | 0.25 |
| Sn | 16.29 | 12.79 | 158 | 0.27 |
| Sb | 18.19 | 13.76 | 161 | 0.33 |
| Ba | 38.16 | 35.84 | 224 | 1.30 |
| Hf | 13.44 | 6.15 | 159 | 0.07 |
| Ta | 10.85 | -0.42 | 147 | 0.20 |
| W | 9.47 | -5.15 | 141 | 0.23 |
| Pt | 9.09 | -6.24 | 139 | -0.51 |
| Au | 10.21 | 0.52 | 144 | -0.04 |
